# Pathogen genomics and phage-based solutions for accurately identifying and controlling *Salmonella* pathogens

**DOI:** 10.3389/fmicb.2023.1166615

**Published:** 2023-04-27

**Authors:** Angela V. Lopez-Garcia, Manal AbuOun, Javier Nunez-Garcia, Janet Y. Nale, Edouard E. Gaylov, Preeda Phothaworn, Chutikarn Sukjoi, Parameth Thiennimitr, Danish J. Malik, Sunee Korbsrisate, Martha R. J. Clokie, Muna F. Anjum

**Affiliations:** ^1^Department of Bacteriology, Animal and Plant Health Agency, Weybridge, United Kingdom; ^2^Department of Veterinary and Animal Science, Scotland's Rural College, Inverness, United Kingdom; ^3^Department of Genetics and Genome Biology, University of Leicester, Leicester, United Kingdom; ^4^Department of Immunology, Faculty of Medicine Siriraj Hospital, Mahidol University, Bangkok, Thailand; ^5^Department of Microbiology, Faculty of Medicine, Chiang Mai University, Chiang Mai, Thailand; ^6^Department of Chemical Engineering, Loughborough University, Loughborough, United Kingdom

**Keywords:** antimicrobial resistance, *Salmonella*, virulence genes, genomics, bacteriophages, serovar

## Abstract

*Salmonella* is a food-borne pathogen often linked to poultry sources, causing gastrointestinal infections in humans, with the numbers of multidrug resistant (MDR) isolates increasing globally. To gain insight into the genomic diversity of common serovars and their potential contribution to disease, we characterized antimicrobial resistance genes, and virulence factors encoded in 88 UK and 55 Thai isolates from poultry; the presence of virulence genes was detected through an extensive virulence determinants database compiled in this study. Long-read sequencing of three MDR isolates, each from a different serovar, was used to explore the links between virulence and resistance. To augment current control methods, we determined the sensitivity of isolates to 22 previously characterized *Salmonella* bacteriophages. Of the 17 serovars included, *Salmonella* Typhimurium and its monophasic variants were the most common, followed by *S.* Enteritidis, *S.* Mbandaka, and *S.* Virchow. Phylogenetic analysis of Typhumurium and monophasic variants showed poultry isolates were generally distinct from pigs. Resistance to sulfamethoxazole and ciprofloxacin was highest in isolates from the UK and Thailand, respectively, with 14–15% of all isolates being MDR. We noted that >90% of MDR isolates were likely to carry virulence genes as diverse as the *srjF*, *lpfD*, *fhuA*, and *stc* operons. Long-read sequencing revealed the presence of global epidemic MDR clones in our dataset, indicating they are possibly widespread in poultry. The clones included MDR ST198 *S*. Kentucky, harboring a *Salmonella* Genomic Island-1 (SGI)-K, European ST34 *S*. 1,4,[5],12:i:-, harboring SGI-4 and mercury-resistance genes, and a *S*. 1,4,12:i:- isolate from the Spanish clone harboring an MDR-plasmid. Testing of all isolates against a panel of bacteriophages showed variable sensitivity to phages, with STW-77 found to be the most effective. STW-77 lysed 37.76% of the isolates, including serovars important for human clinical infections: *S*. Enteritidis (80.95%), *S*. Typhimurium (66.67%), *S*. 1,4,[5],12:i:- (83.3%), and *S*. 1,4,12: i:- (71.43%). Therefore, our study revealed that combining genomics and phage sensitivity assays is promising for accurately identifying and providing biocontrols for *Salmonella* to prevent its dissemination in poultry flocks and through the food chain to cause infections in humans.

## Introduction

Non-typhoidal *Salmonella* (NTS), one of the principal causes of limiting diarrheal diseases in humans due to the presence of a myriad of virulence determinants, is responsible for approximately 155,000 deaths annually (Pan et al., 2009; Majowicz et al., 2010; Eguale et al., 2015; Balasubramanian et al., 2019; Nale et al., 2021). It is estimated that 93.8 million cases of gastroenteritis caused by *Salmonella* infection occur worldwide each year (Majowicz et al., 2010), and contaminated poultry products are one of the main routes of transmission to humans (Foley et al., 2008; Nale et al., 2021).

Problems with *Salmonella* control are a major concern and cause significant economic losses in the global poultry sector (Wang et al., 2018; Li et al., 2020). Asymptomatic chickens cannot be easily identified and isolated, and as a result, they shed *Salmonella* from their faeces in the environment and this contributes to the spread of *Salmonella* in poultry and the contamination of poultry products (Kogut and Arsenault, 2017). *Salmonella* can also reach the production system by a number of other routes, including infected feed, water, litter, or live vectors, and humans *via* contaminated footwear or equipment (Wales et al., 2010; Machado Junior et al., 2020). Thus, each stage of processing from farm to fork poses a danger of *Salmonella* contamination and infection (Akil and Ahmad, 2019).

As a result, preventing and controlling this illness in humans is important for interrupting the cycle of infection within the food chain before the products are accessible for consumption (Nale et al., 2021). According to a USDA report, global Thai chicken meat exports totaled 519,228 tons in the first half of 2021 (Service USDoAFA, 2021). Additionally, the volume of poultry meat produced in the UK was on average 150,000 tons of carcase weight in 2021 and 2022 (Department for Environment Food and Rural Affairs, 2022). Therefore, food quality and food safety are vital for Thai and UK inhabitants, as well as those throughout the world.

The susceptibility of *Salmonella* to different antimicrobial classes has decreased over the years. In the UK, a reduction of AMR susceptibility in poultry isolates was observed from 2018 (84%) to 2021 (approximately 70%); despite the use of antimicrobials within the poultry sector decreasing in this period (Veterinary Medicines Directorate, 2021). Although national data on antimicrobial use in the poultry sector in Thailand are currently not available, a survey of eight Thai farms in 2016 noted that 161 tons of antibiotics were administered to Thai chickens raised for meat (Wongsuvan et al., 2018). Importantly, multidrug resistant (MDR) *Salmonella* isolates have been detected in poultry (Card et al., 2016); *Salmonella* resistant to colistin, a last-resort antibiotic used for treating MDR bacteria in humans, has been reported in livestock (Duggett et al., 2016, 2018).

The most critical evolution of AMR occurs in Gram-negative bacteria, mainly in *Enterobacteriaceae* (America IDSo, 2011; Lynch et al., 2013), in which AMR genes are transferred by vertical and horizontal transmission (Vikesland et al., 2019). Several AMR-harboring *Salmonella enterica* clades have emerged and are disseminating worldwide, including the global *S*. Kentucky MDR-ST198 clone, which harbors *Salmonella* genomic island (SGI) 1 (SGI1-K variant; Hawkey et al., 2019). In the preceding two decades, three other significant *S*. Typhimurium and its monophasic variant clones have been spreading: the European, Spanish and US clones (Nale et al., 2021). The *S*. 1,4,[5],12:i:- ST34 European clone encodes a chromosomal element that confers resistance to four antimicrobials (Petrovska et al., 2016). The Spanish *S*. 1,4,12:i:- ST19 clone harbors a plasmid that provides resistance to seven antimicrobials (Antunes et al., 2011; Duggett et al., 2016), while the US ST19 clone is rarely resistant to antimicrobials (Cadel-Six et al., 2021). Combating the rise of antibiotic resistance is one of the most critical issues facing our society today, and the interdependence between the control and transmission of antimicrobial resistance and virulence may need to be considered to achieve significant improvements in the treatment of bacterial infections (Schroeder et al., 2017).

Bacteriophages (phages) are natural viruses for bacteria, and, as such, are a viable alternative to current antimicrobials (Salmond and Fineran, 2015; Czaplewski et al., 2016). Phages have been shown to lyse MDR *Salmonella* isolates, suggesting their potential role in the control of these pathogens in the food chain (Jung et al., 2017; Thanki et al., 2019; AbuOun et al., 2020; Nale et al., 2021). Owing to an increase in AMR-harboring bacteria and a paucity of new antimicrobials, research into the therapeutic use of lytic phages (also known as “phage therapy”) has been increasing at an exponential rate (Nobrega et al., 2015) and there are already some phage cocktails presenting promising lysis activity against *Salmonella* (Nale et al., 2021).

In this study, we characterized antimicrobial and virulence factors of a collection of 143 *Salmonella* isolates from poultry farms in the UK and Thailand, where farming systems differ as the prophylactic use of antimicrobials and colistin, a critically important antimicrobial for human therapy, is banned in the UK^1^; there is variable use of antimicrobials on poultry farms in Thailand (Wongsuvan et al., 2018). Genomic analysis explored differences and similarities in the AMR and virulence profiles of the isolates from both countries, which possibly reflect different husbandry practices and, importantly, the use of antimicrobials. The potential for the dissemination of these genes was assessed by the detailed genomic characterization of mobile genetic elements within selected global MDR clones identified within the dataset. In addition, the characterized *Salmonella* isolates were used as a reference panel to test the lytic activity of a collection of phages to establish potential biocontrols.

## Materials and methods

### Bacterial isolates and bacteriophages

A total of 143 *Salmonella* isolates purified from poultry faeces collected on farms were used in this study; the date of isolation, serovar, phage-type (where available), and country of origin are provided in Supplementary Table S1. Thai isolates were sent from Thailand in 2019 and were samples of convenience. The UK (*n* = 88) isolates were acquired from diagnostic submissions or the *Salmonella* National Control Program in the UK between 2003 and 2019, which is held in the APHA collection. It included isolates randomly selected from the most common serovars collected in that period, as well as isolates randomly selected from any additional serovars represented by the Thai isolates, to enable cross comparison. All isolates were serotyped according to the White–Kauffmann–Le Minor scheme (9th Edition; Grimont and Weill, 2007) at the APHA *Salmonella* reference laboratory.

Twenty-two *Salmonella* phages, the biological properties of which have been extensively reported (Phothaworn et al., 2020; Nale et al., 2021; Sukjoi et al., 2022; Nale et al., 2023), were tested. Twenty (SPFM9, SPFM11, SPFM17, SPFM4, SPFM2, SPFM19, SPFM14, SPFM10, SPFM12, SPFM13, SPFM20, SPFM1, SPFM3, SPFM16, SPFM15, SPFM7, SPFM6, SPFM8, SPFM21, and SPFM5) were previously isolated and characterized in a UK laboratory, while two (STW-77 and SEW-109) were previously isolated and characterized in Thailand (Thanki et al., 2019; Phothaworn et al., 2020; Nale et al., 2021).

### Minimum inhibitory concentration determination

The MIC (minimum inhibitory concentration) values for all the isolates were determined using the sensititre broth microdilution method (Thermo Fisher Scientific, n.d.). All isolates were screened against ampicillin, azithromycin, cefotaxime, ceftazidime, chloramphenicol, ciprofloxacin, colistin, gentamicin, meropenem, nalidixic acid, sulfamethoxazole, tetracycline, tigecycline, and trimethoprim (EUVSEC plates, Thermofisher). *Escherichia coli* ATCC 25922 was used as a control. MIC values were mainly interpreted using the EUCAST ECOFFs (EUCAST, 2020), except for tigecycline, for which cutoff values were interpreted according to the EFSA report (Cadel-Six et al., 2021).

### DNA extraction and short-read sequencing

For all 143 isolates, DNA was extracted using a MagMax core nucleic acid purification kit and a Kingfisher Flex system (Thermo Fisher, USA; Duggett et al., 2020), and short-read whole-genome sequenced (WGS) using the Illumina NextSeq platform, as described previously (Duggett et al., 2018), at the APHA Central Sequencing Unit. *Salmonella* genome assemblies were generated using Unicycler version 0.4.4 (Wick et al., 2017). The presence of acquired AMR genes in the isolate WGS was determined by mapping unassembled reads using the APHA SeqFinder V1.2 pipeline (Stubberfield et al., 2019), which was used to predict AMR phenotypes with >98% accuracy. An AMR gene was considered present if there was ≥90% of the gene mapped to the reference gene present in the APHA SeqFinder database, allowing between 1 and 10 non-synonymous SNPs, and Abricate was used to corroborate any ambiguity, as previously described (AbuOun et al., 2021; Nunez-Garcia et al., 2022). MegAlign Pro - DNASTAR Lasergene (DNASTAR Inc., Madison, US) was used to perform gene alignments with *Salmonella* Typhimurium LT2 (accession number AE006468.2) as the reference. This was performed to identify the SNPs associated with fluoroquinolone resistance in chromosomal genes/regions, *gyrA*, *parC*, *parE*, and *ampC*. SNPs previously identified as conferring fluoroquinolone resistance were included in the APHA SeqFinder pipeline (AbuOun et al., 2020).

A *Salmonella* virulence gene database was constructed to detect new genes/putative proteins within the 17 *Salmonella* pathogenicity islands (SPI; Kombade and Kaur, 2021) and add to the current virulence factors database (VFDB; Chen et al., 2005), which has 174 genes. The APHA database included 1,849 genes and putative proteins obtained from the published literature for analysis in the APHA SeqFinder. The sequence of the genes and putative proteins spanned a range of virulence and regulatory associated functions, including adhesion (*n* = 90), plasmid related (*n* = 710), SPIs (*n* = 791), phage related (*n* = 191), toxin (*n* = 3), regulation factors (*n* = 2), *Salmonella* Genetic Island-associated genes (*n* = 48), and other virulence-associated genes (*n* = 14). The presence of virulence genes was determined using the APHA SeqFinder and Abricate with a query gene coverage of >80% and gene identity of >70%. MLST (multi-locus sequence typing) was used to determine the isolates sequence type (ST; Larsen et al., 2012). Blast Ring Image Generator (BRIG) version 0.95 (Alikhan et al., 2011) was used to identify the presence of *Salmonella* Genomic Island (SGI1-K).

### Long-read sequencing

Three MDR isolates (BL700, BL708, and BL661) with interesting AMR and virulence profiles were selected for long-read sequencing. A PDQex kit from ZyGEM NZ Ltd. was used for DNA extraction, following the manufacturer’s instructions for Gram-negative colonies and biofilm. The protocol was modified in consensus with the manufacturer extending the lysis phase at 75°C for an additional 10 min. DNA concentrations were quantified using a Qubit 3.0 fluorometer (Qubit dsDNA Broad Range Assay Kit, Life Technologies; Thermo Fisher, USA) and DNA fragment lengths were determined using agarose gel electrophoresis. Isolate DNA was long-read sequenced using the Oxford Nanopore MinION (Nunez-Garcia et al., 2022). The quality of the sequences was measured in Nanoplot (De Coster et al., 2018). Long-read assembly was performed using Unicycler and a hybrid assembly strategy (Nunez-Garcia et al., 2022). As already stated, the APHA SeqFinder in combination with the Abricate tool using the APHA SeqFinder AMR database was used to identify the presence of AMR genes. Plasmid and AMR gene locations were given preliminary annotation using Prokka (Seemann, 2014) and visualized using EasyFig (Sullivan et al., 2011) and BRIG (Alikhan et al., 2011). The raw reads for isolates from this dataset, including long reads, are available in the NCBI nucleotide archive under submission ID: SUB12515972 and project ID: PRJNA919003.

### Phylogenetic and SNP analysis

Snippy version v4.6.0 (Seemann, 2015) was used to detect SNPs in the core genome of *S*. Kentucky isolates BL700 and BL800, and two *S*. Kentucky MDR ST198 isolates from earlier research [SAMN08784244 and SAMN08784253; (Hawkey et al., 2019)], aligning them against the reference 201001922 (CP028357). Additionally, *S*. Typhimurium and monophasic *S*. Typhimurium isolates BL665, BL667, BL691, BL708, BL709, BL711, BL754, and BL757, along with two MDR ST34 monophasic *S*. Typhimurium isolates from previous studies, S01861-18 (accession number SAMEA5757827) and 12CEB4916SAL (accession number SAMEA5206613; Cadel-Six et al., 2021), were aligned against the reference LT2 (AE006468) using the same tool.

A rooted maximum-likelihood tree based on SNPs in the core-genome of all *S*. Typhimurium isolates and its variants in our collection, and other isolates of the same serotypes available in Enterobase (Wen et al., 2020; Supplementary Table S2) was bootstrapped using RAxML-NG (Kozlov et al., 2019) under the L8 + G8 + F model and using SL1344 (accession number NC_016810.1) as a reference. The phylogenetic tree was annotated and visualized with iTOL (Letunic and Bork, 2019). Supplementary Table S3 provides details of the number of SNP differences between isolates included in this analysis with respect to the reference strain SL1344, in the core genome, used to construct the phylogenetic tree.

### Phage host range lytic assays

The host range of phages was investigated by adding 10 μl volumes of 10^8^ PFU/ml volumes of lysates to confluently grown bacterial isolates and incubating plates aerobically for 18 h at 37°C. Three biological and technical duplicates of plates were evaluated for bacterial lysis as described previously (Nale et al., 2021).

## Results

A collection of *Salmonella* isolates from poultry in the UK (*n* = 88) and Thailand (*n* = 55) from 2003 and 2019 were serotyped using both conventional antigen-based and *in silico* serotyping using WGS to identify their serovars (Supplementary Table S1). The results indicated that although most isolates belonged to serovars *S.* Enteritidis, *S*. Virchow, *S*. Mbandaka, or *S.* Typhimurium, several other serovars were also identified (Supplementary Table S4).

**Figure 1:**
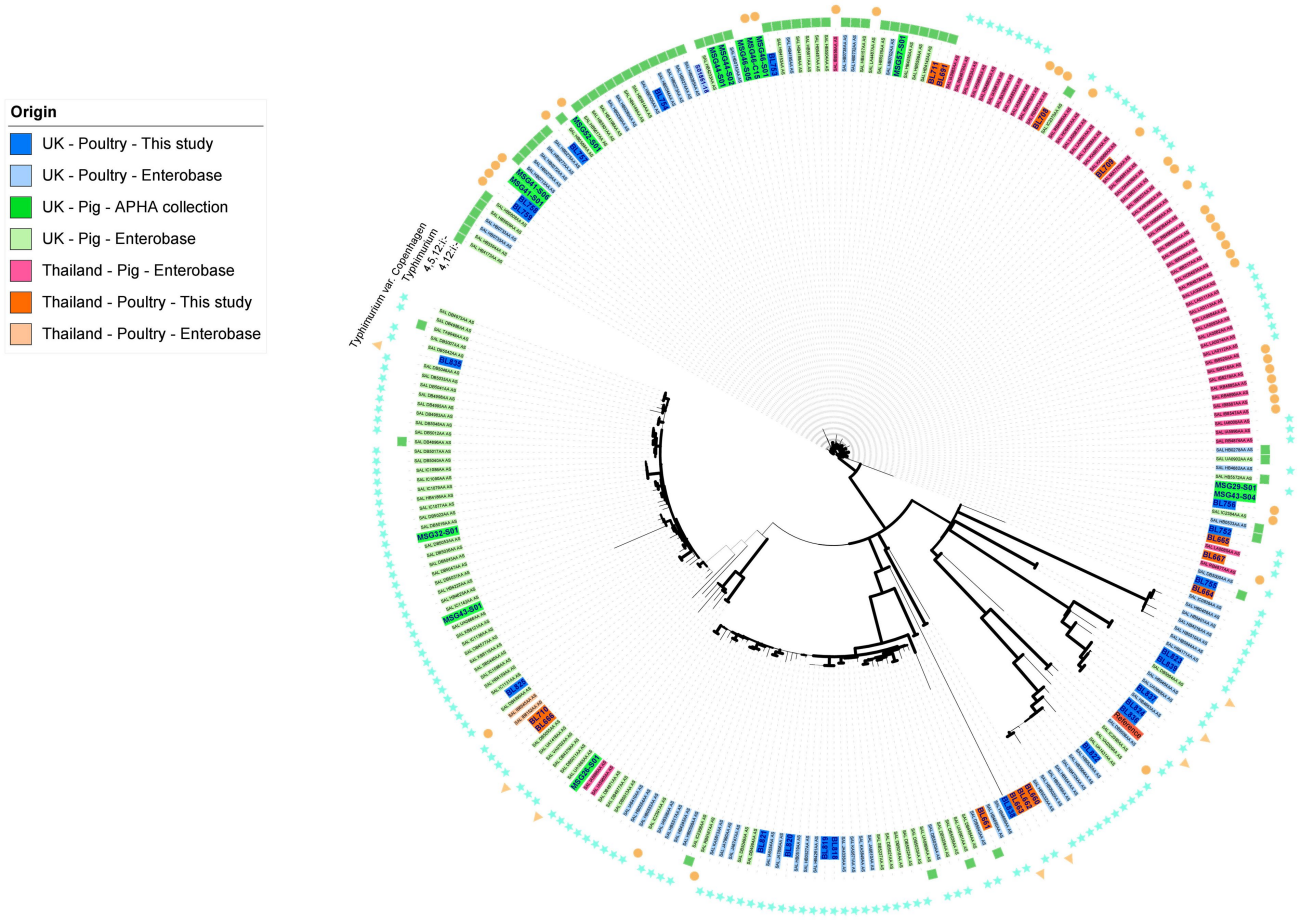
Maximum likelihood tree of *S*. Typhimurium, *S*. Typhimurium variant Copenhagen, *S*. 1,4,[5],12:i:-, and *S*. 1,4,12:i:- isolates based on SNPs in the core-genome. The tree was constructed in RaxML-NG using 17,263 single nucleotide polymorphisms (SNP). The isolate names have been colored based on their country, source, and database of origin. Dark blue corresponds to sequences from the APHA isolate collection belonging to the UK poultry included in this study (*n* = 21); dark green represents archived APHA UK pig isolates (*n* = 14); dark orange represents isolates from the study collection belonging to Thai poultry (*n* = 13); light blue represents Enterobase isolates belonging to UK poultry (*n* = 64); light green represents Enterobase isolates belonging to UK pigs (*n* = 96); light orange represents Enterobase isolates belonging to poultry from Thailand (*n* = 2); and light pink represents Enterobase isolates from pigs in Thailand (*n* = 57). Branches are colored in black for bootstrap values of >70 to provide confidence. The outer ring indicates the serovar of the isolates.

To ensure *S*. Typhimurium and its serovariants included in this study, which together was the most common and clinically important serovar of the collection, were representative of their respective countries and animal host, a core genome SNP-based maximum-likelihood tree (Figure 1) was constructed based on SNP distance (Supplementary Table S3). It included all isolates from serovar *S*. Typhimurium and variants *S*. 1,4,[5],12:i:-, *S*. 1,4,12:i:-, and *S*. Typhimurium variant Copenhagen from our collection, as well as poultry isolates from these serovars present in Enterobase (Wen et al., 2020) from the UK and Thailand; we also included a handful of isolates from pigs from the UK present in the APHA archive and Enterobase. The core genome for *S*. Typhimurium and variant isolates was 17,263 base pairs and the average size of the *Salmonella* genome for isolates sequenced in this study was 4.6 Mb. In the *S*. Typhimurium phylogenetic tree (Figure 1), the main node was subdivided into several clusters; the bootstrap values (>70) provided confidence in the division of the clades and nodes. The isolates were primarily clustered in agreement with their host animal, although for some clusters, isolates of different sources were interspersed within clades; the pig isolates from UK and Thailand separated into distinct clusters. The maximum number of SNPs between the collected isolates was 12,620 bp (Supplementary Table S3), reflecting the genetic diversity of the subpopulations of *S*. Typhimurium and variants that have emerged in Thailand and the UK in this period.

A phylogenetic tree was built using the genomes of *S.* Enteritidis isolates from this study and those available in Enterobase (UK poultry, *n* = 201; UK pigs, *n* = 4; Thailand, none available), as this serovar is most often associated with human cases. It showed Enteritidis isolates were genetically very diverse, with the core genome <5,000 bp in size (data not shown), making it difficult to ascertain how representative our panel of isolates were of each country. The numbers of *S.* Mbandaka and *S.* Virchow genomes present in Enterobase from poultry from the UK and Thailand were too low to build a phylogenetic tree to obtain an idea of their representation within each country.

### Antimicrobial resistance characterization

Antimicrobial resistance (AMR) characterization was performed on the collection of isolates to compare AMR profiles between the UK and Thai isolates. The 143 isolates were genotypically and phenotypically characterized using the APHA SeqFinder pipeline and sensititre broth microdilution methods, respectively (see Materials and Methods). The correlation between genotype and phenotype was >99% (Supplementary Table S5) for *Salmonella* isolates, so only the genotypic data has been discussed in detail in this paper. The APHA SeqFinder pipeline identified 20 different AMR genes (Table 1) in the UK and Thai isolates. The most common AMR gene in the UK and Thai isolates was *bla_TEM-1b_*, which was present in 11 of 88 (12.50%) and 13 of 55 (23.64%) isolates, respectively. Approximately 27.27% of the Thai isolates (15 of 55) harbored mutations in *gyrA*, conferring fluoroquinolone resistance, which was more than five times the number (4 of 88, 4.55%) from the UK isolates in the collection (Table 1).

**Table 1:** AMR genotypes of 143 isolates identified by the APHA Seqfinder.

Classes	Antimicrobial	Genes	UK (n isolates)	Percentage	Thai (n isolates)	Percentage
Penicillin	Ampicillin	*bla_CARB-2_*	4 of 88	4.55%	0 of 55	0%
		*bla_TEM-135_*	0 of 88	0%	3 of 55	5.45%
		*bla_TEM-1b_*	11 of 88	12.50%	13 of 55	23.64%
		*bla_TEM-1D_*	1 of 88	1.14%	0 of 55	0%
Macrolides	Azithromycin	*mphB*	1 of 88	1.14%	0 of 55	0%
Phenicol	Chloramphenicol	*cmlA1*	4 of 88	4.55%	1 of 55	1.82%
		*floR*	4 of 88	4.55%	0 of 55	0%
Fluoroquinolone	Ciprofloxacin	*mutation gyrA*	4 of 88	4.55%	15 of 55	27.27%
		*qnrS1*	0 of 88	0%	4 of 55	7.27%
	Nalidixic acid	*mutation gyrA*	4 of 88	4.55%	15 of 55	27.27%
Aminoglycoside	Gentamicin	*aac(3)-Id*	1 of 88	1.14%	0 of 55	0%
		*aac(3)-IVa*	2 of 88	2.27%	1 of 55	1.82%
	Streptomycin	*strA*	7 of 88	7.95%	7 of 55	12.73%
		*strB*	9 of 88	10.23%	7 of 55	12.73%
Sulfonamide	Sulfamethoxazole	*sul1*	4 of 88	4.55%	1 of 55	1.18%
		*sul2*	6 of 88	6.82%	8 of 55	14.55%
		*sul3*	7 of 88	7.95%	5 of 55	9.09%
Tetracycline	Tetracycline	*tetA(B)*	7 of 88	7.95%	5 of 55	9.09%
		*tet(A)*	6 of 88	6.82%	2 of 55	3.64%
		*tet(G)*	4 of 88	4.55%	0 of 55	0%
		*tet(M)*	0 of 88	0%	1 of 55	1.82%
Diaminopyrimidine	Trimethoprim	*dfrA12*	4 of 88	4.55%	1 of 55	1.82%

AMR genes were grouped according to antimicrobial classes, and they are shown alongside the percentage of isolates per country harboring the AMR gene.

**Figure 2:**
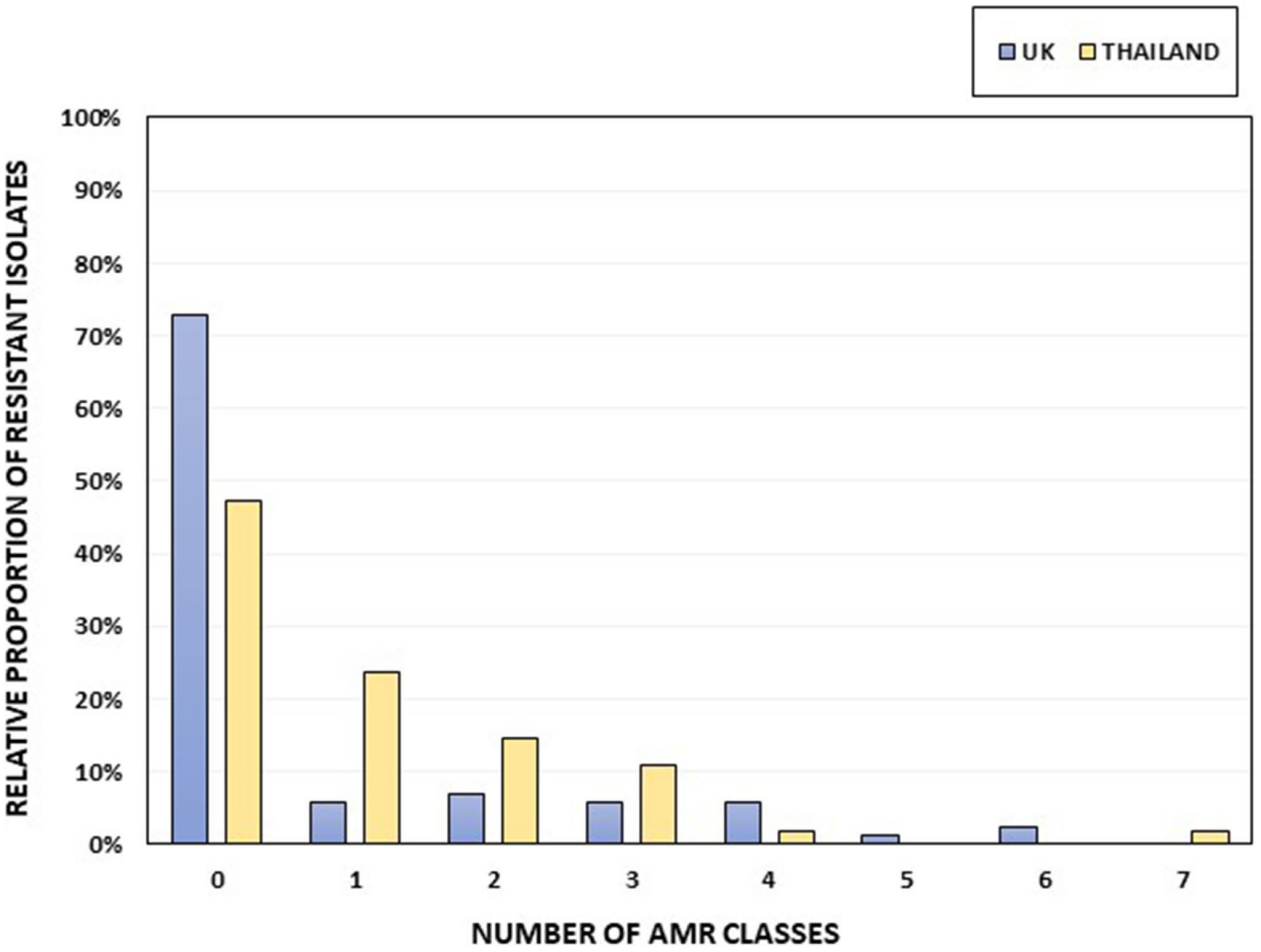
The percentage of isolates per country harboring AMR genes, grouped according to resistance to different AMR classes. Thai isolates are shown in yellow and UK isolates in blue. The total numbers of UK and Thai isolates were 88 and 55, respectively.

Thirteen of the 88 UK *Salmonella* isolates (14.77%) and eight of the 55 Thai isolates (14.55%; Figure 2) showed an MDR genotype (resistance to at least three different antimicrobial classes). One Thai isolate, serotyped as *S*. 1,4,12:i:-, was resistant to seven antimicrobial classes (ampicillin, chloramphenicol, ciprofloxacin-nalidixic acid, gentamicin, sulfamethoxazole, tetracycline, and trimethoprim). Two UK isolates classified as *S*. 1,4,[5],12:i:- showed resistance to six antimicrobial classes (tetracycline, gentamicin-streptomycin, chloramphenicol, sulfamethoxazole, ampicillin, and trimethoprim). The serovars with the highest number of MDR isolates within our collection were *S*. 1,4,[5],12:i:- (5 of 6, 83.33%), *S*. Kentucky (2 of 4; 50%), *S*. Typhimurium (8 of 15, 53.33%), and *S*. 1,4,12:i:- (3 of 7, 42.85%; Supplementary Table S6). No AMR genes were detected in 72.73% of the UK isolates (64 of 88), whereas 47.27% (26 of 55) of the Thai isolates were susceptible to all the included antimicrobials (Figure 2; Supplementary Table S6).

*S*. Mbandaka, *S.* Bovismorbificans, and *S*. Agona isolates were sensitive to all antimicrobials tested (Supplementary Table S6). For UK isolates (Table 2), the highest percentage of AMR was for sulfamethoxazole (19.32%), followed by ampicillin (18.18%) and tetracycline (17.05%). For Thai isolates (Table 2), the highest percentages of resistance were to ciprofloxacin (34.55%), ampicillin (29.09%), and nalidixic acid (27.27%). In addition, all UK isolates were sensitive to cefotaxime, ceftazidime, colistin, meropenem, and tigecycline. No resistance was detected to azithromycin, cefotaxime, ceftazidime, colistin, meropenem, and tigecycline for the Thai isolates.

**Table 2:** The percentage of isolates per country harboring genes conferring resistance to an antimicrobial.

Antimicrobial	UK	Thailand
Ampicillin	16 of 88 (18.18%)	16 of 55 (29.09%)
Azithromycin	1 of 88 (1.14%)	0%
Cefotaxime	0%	0%
Ceftazidime	0%	0%
Chloramphenicol	8 of 88 (9.09%)	1 of 55 (1.82%)
Ciprofloxacin	4 of 88 (4.55%)	19 of 55 (34.55%)
Colistin	0%	0%
Gentamicin	3 of 88 (3.41%)	1 of 55 (1.82%)
Meropenem	0%	0%
Nalidixic acid	4 of 88 (4.55%)	15 of 55 (27.27%)
Sulfamethoxazole	17 of 88 (19.32%)	12 of 55 (21.82%)
Tetracycline	15 of 88 (17.05%)	8 of 55 (14.55%)
Tigecycline	0%	0%
Trimethoprim	4 of 88 (4.55%)	1 of 55 (1.82%)
Streptomycin	9 of 88 (10.23%)	7 of 55 (12.73%)

The percentage AMR detected was based only on genotypes.

### Virulence characterization

The pathogenicity of *Salmonella* is based on the presence of virulence determinants. Therefore, an extensive database containing sequences of 1,849 virulence genes and putative proteins associated with 17 *Salmonella* pathogenicity islands (SPI; Kombade and Kaur, 2021) was designed and run through the APHA SeqFinder pipeline (AbuOun et al., 2021). This database is an improvement on the public domain virulence factors database (VFDB; Chen et al., 2005), which has 174 genes mainly focused on the five core SPIs. The aim was to determine the association of virulence profiles in isolates with factors such as AMR (MDR; non-MDR but harboring resistance; fully sensitive), serovar, and country (UK or Thailand).

**Figure 3:**
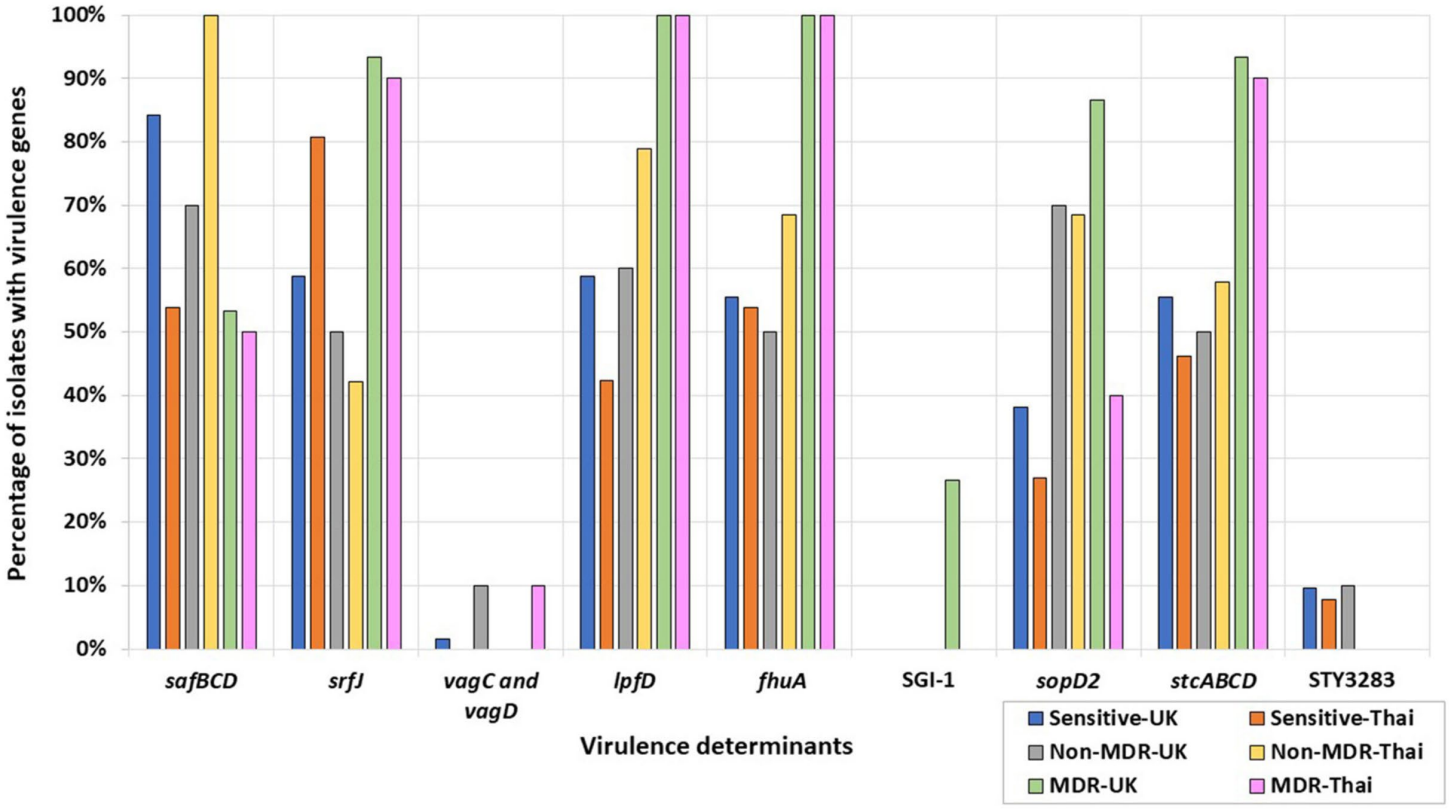
Percentages of UK and Thai MDR, non-MDR, and sensitive isolates harboring virulence determinants. MDR isolates from the UK (green), non-MDR isolates from the UK (grey), sensitive isolates from the UK (blue), MDR isolates from Thailand (pink), non-MDR isolates from Thailand (yellow), and sensitive isolates from Thailand (orange) have been included.

Some MDR isolates were more likely to harbor specific virulence determinants or genes (Figure 3) than isolates with resistance to less than three antimicrobial classes, i.e., non-MDR or fully sensitive isolates. Conversely, several virulence determinants were more likely to be present in non-MDR and sensitive isolates than in MDR isolates (Figure 3). For example, the *Salmonella* plasmid harboring virulence genes *vagC* and *vagD* (Pullinger and Lax, 1992) was identified only in the MDR Thai *S*. 1,4,12:i:- isolate but was absent from the remaining UK and Thai *S*. 1,4,12:i:- isolates (Supplementary Table S7). When considering *Salmonella* serovars more widely, these genes were identified in 10% of the Thai MDR isolates and 10% of the UK non-MDR isolates, respectively, but only 1.59% of fully sensitive isolates from the UK. They were not detected in MDR-UK and non-MDR/fully sensitive Thai isolates (Figure 3).

All MDR *S*. Kentucky isolates from the UK and Thailand harbored the Saf pili operon genes, *safBCD* (Zeng et al., 2017), the T3SS-2 effector *srfJ* gene (Cordero-Alba et al., 2012), the bacteriophage receptor and ferric iron transporter *fhuA* (Wang et al., 2018), and the *lpfD* gene encoding the Lpf fimbrial adhesin tip (Cohen et al., 2021). The remaining *S*. Kentucky in the collection were sensitive isolates from the UK and did not harbor these genes (Supplementary Table S7). More widely, *srfJ*, *fhuA*, and *lpfD* were detected in higher percentages in the UK and Thai MDR isolates, while the presence of the *safBCD* operon was higher in non-MDR and sensitive Thai and UK isolates (Figure 3).

The fimbrial operon *stcABCD* (Holman et al., 2018) and the STY3283 bacteriocin immunity protein (Pawar et al., 2017) were present in all the UK sensitive *S*. Kentucky isolates (Supplementary Table S7). More widely, STY3283 was also present in 9.52% of the UK sensitive isolates and 7.69% of the Thai sensitive isolates. The *stc* fimbrial operon was also present from isolates of the wider collection, differing according to AMR category: 55.56 and 46.15%, respectively, of the UK and Thai sensitive isolates; 50 and 57.89%, respectively, of the non-MDR UK and Thai isolates; and 93.33 and 90.00%, respectively, of the MDR UK and Thai isolates (Figure 3).

The SPI-1 gene *sopD2* (Figueiredo et al., 2015), identified in all MDR UK *S*. Typhimurium isolates, was not identified in any UK *S*. Typhimurium sensitive isolates. By contrast, it was inconsistently present in isolates of Thai *S*. Typhimurium. More widely, the gene was generally present at a higher percentage in UK serovars and was detected in 86% of the MDR, 70% of the non-MDR, and 38% of the fully sensitive UK isolates; by contrast, 40% of the MDR, 68% of the non-MDR, and 27% of the fully sensitive Thai isolates harbored this gene (Figure 3). *Salmonella* Genomic Island 1 (SGI-1) was only found in four out of five UK MDR *S*. Typhimurium strains, all of which were phage type DT104. The only UK MDR *S*. Typhimurium isolate without SGI-1 was a phage type DT193 isolate (Supplementary Table S7; Figure 3).

### Characterization of MDR isolates with distinct virulence profiles

We performed long-read sequencing and used the hybrid assemblies to resolve the genomes of three isolates, which showed both interesting MDR and virulence profiles; they were from serovars *S*. Kentucky (BL700; Thailand), *S*. 1,4,[5],12:i:- (BL708; Thailand), and *S.* 1,4,12:i:- (BL661; Thailand).

1. a. *S*. Kentucky (BL700; Thailand)

**Figure 4:**
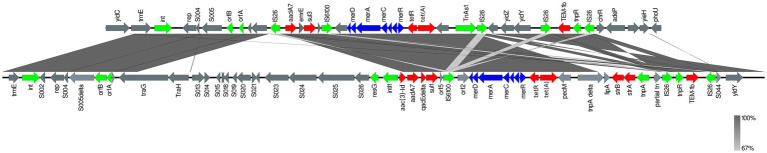
Comparison of SGI1-K (accession number AY463797.8; bottom line) with SGI1-K from BL700 (top line). Genes are represented by arrows pointing in the direction of transcription and different colors based on predicted function; transposase, recombinase, and resolvase are indicated by green arrows; AMR genes are indicated in red; mercury resistance genes are indicated in blue; and open reading frames of unknown function are indicated in grey. The grey shading connecting regions of nucleotides indicates sequence identity, ranging from 80 to 100% (see scale).

**Figure 5:**
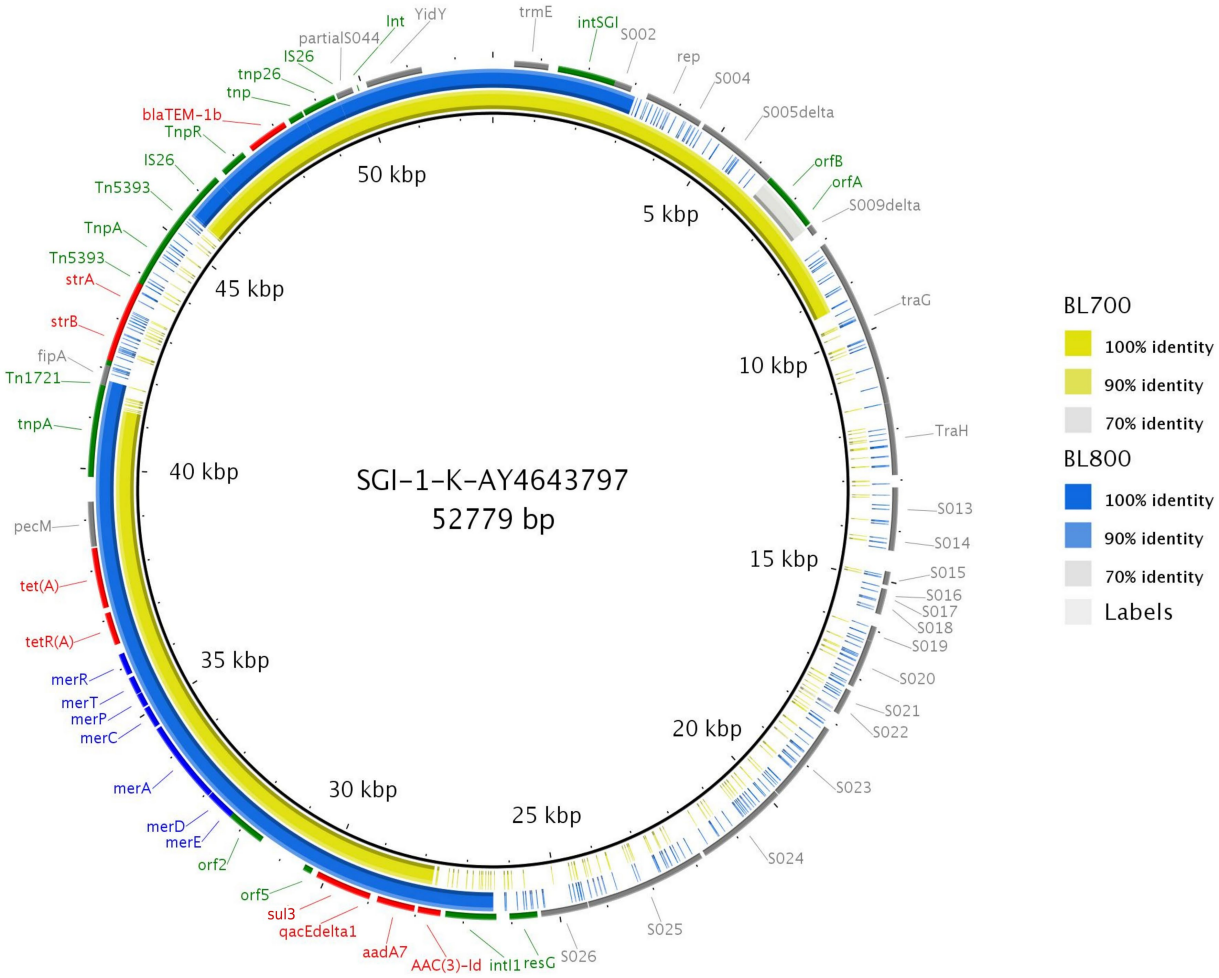
Genome comparison of SGI-1-K present in two MDR *S.* Kentucky isolates through the alignment of SGI-1-K present in BL700 and BL800 to the reference (accession number AY463797.8). Solid lines denote 100% sequence identity, with yellow indicating BL700 and blue indicating BL800; low or no sequence identity are shown in grey or as gaps. Genes flanking SGI-1-K in the reference (grey), AMR (red), mercury resistance (blue), transposon, resolvase, and integron genes (green) are shown.

Using the resolved genome in BL700, we located all transferable AMR genes [*aadA7*, *bla_TEM-1b_*, *sul3*, and *tet(A)*] in a 32,805 bp region within the chromosome. A BlastN query indicated high sequence identity to *Salmonella* Genomic Island (SGI)-1-K (accession number AY4643797.8), which also carries a mercury resistance region. The SGI1-K present in BL700 showed a deletion in its backbone spanning from the truncated conjugal transfer protein *traG* to the *intI1* gene. In addition, deletions in three AMR genes [*aac(3)-Id*, *strB*, and *strA*] were noted in BL700 SGI-K (Figure 4). Another isolate (UK *S*. Kentucky BL800) from our panel also harbored SGI1-K. Although it harbored *aadA7*, *bla_TEM-1b_*, *sul3*, *tet(A)*, and *aac(3)-Id*, we detected several deletions in its backbone, which were attributed to IS26-mediated insertion and deletion events (Figure 5). The sequence type (ST) of both isolates was ST198. As a global lineage of MDR *S*. Kentucky ST198 (Hawkey et al., 2019) has been reported previously, we mapped BL800, BL700, and other MDR *S.* Kentucky ST198 clone sequences present in NCBI to the draft *S.* Kentucky reference (accession number CP028357.1). BL700 was 12 SNPs from a 2012 human Vietnamese isolate (201207374; SAMN08784244) and BL800 was 16 SNPs from a 2008 Moroccan seafood isolate (08-015; SAMN08784253), indicating both isolates were part of the global MDR *S.* Kentucky ST198 lineage. The virulence genes *safBCD*, *srfJ*, *lpfD*, and *fhuA* identified in the *S*. Kentucky MDR clones were not colocated with SGI-1-K.

1. b. *S*. 1,4,[5],12:i:- (BL708; Thailand)

From the resolved genome of MDR isolate BL708 *S.* 1,4,[5],12:i:-, we identified SGI-4 in the chromosome with genes showing resistance to copper, arsenic, mercury, and antimicrobials [*bla_TEM-1B_, sul2, tetA(B), strA, strB*]. These AMR genes were also identified in seven other ST34 isolates included in this study from serovars *S*. Typhimurium, *S*. 1,4,12:i:-, and *S*. 1,4,[5],12:i:- (Table 3).

**Table 3:** Identification of mercury resistance genes and SGI-4 in ST34 S.

Id	Serotype	Country	ST	Year	Source	Mercury resistance genes	AMR profile	SGI-4		SNP difference	
								Arsenic resistance genes	Copper and Silver resistance	S01861-18	12CEB4916SAL
BL665	Typhimurium	Thailand	34	18/19	Poultry	✓	ASSuT	✓	✓	45	39
BL667	Typhimurium	Thailand	34	18/19	Poultry	✓	ASSuT	✓	✓	44	39
BL691	1,4,12:i:-	Thailand	34	18/19	Poultry	✓	ASSu-	✓	✓	51	46
BL708	1,4,[5],12:i:-	Thailand	34	18/19	Poultry	✓	ASSuT	✓	✓	44	38
BL709	1,4,[5],12:i:-	Thailand	34	18/19	Poultry	✓	ASSuT	✓	✓	47	41
BL711	1,4,12:i:-	Thailand	34	18/19	Poultry	✓	ASSu-	✓	✓	45	40
BL754	1,4,12:i:-	UK	34	2018	Poultry	✓	ASSuT	✓	✓	38	48
BL757	1,4,[5],12:i:-	UK	34	2013	Poultry	✓	ASSuT	✓	✓	32	42

Antimicrobial abbreviation: ampicillin (A), sulfamethoxazole (Su), Streptomycin (S), and tetracycline (T). Typhimurium and monophasic variants. The SNP distance in the core genome between ST34 isolates from this study and previously reported ST34 European clones (S01861-18 and 12CEB4916SAL) is shown.

A European lineage of *S*. 1,4,[5],12:i:- ST34, harboring SGI-4, mercury, and ASSuT resistance genes, has been reported previously (Petrovska et al., 2016; Cadel-Six et al., 2021). The genetic relatedness between BL708 and two previously reported isolates (S01861-18 and 12CEB4916SAL) was studied. Both S01861-18 and 12CEB4916SAL belonged to the main clade for the *S*. 1,4,[5],12:i:- ST34 (clade A) global lineage, in which the average distance between isolates was 41 SNPs (Cadel-Six et al., 2021). The eight UK and Thai ST34s in our panel were approximately 41 SNPs apart from 12CEB4916SAL and approximately 43 SNPs apart from S01861-18, indicating that these isolates were part of the same lineage (Table 3). None of the virulence genes identified were colocated with SGI-4.

1. c. *S*. 1,4,12:i:- (BL661; Thailand)

**Figure 6:**
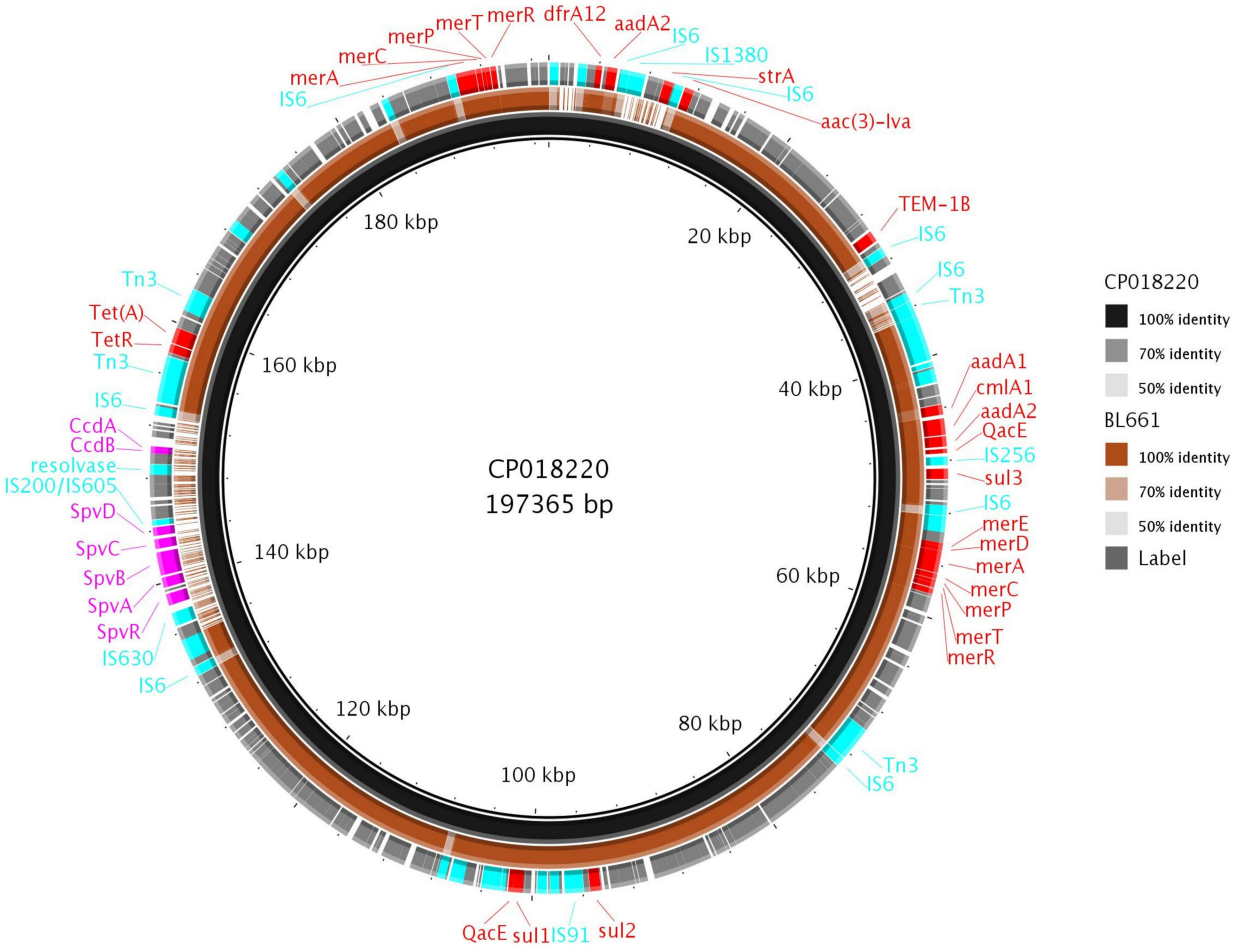
Comparison of pBL661 with the reference plasmid CP018220. The outer ring shows the annotated genes, with 100% identity (solid brown), as well as low (light brown or grey) or no sequence identity (gaps).

The Thai isolate BL661 *S*. 1,4,12:i:- showed resistance to seven AMR classes and was the most resistant *Salmonella* in the collection. The resolved genome revealed that the AMR [*blaTEM-1b*, *cmlA1*, *aac(3)-Iva*, *aadA1, aadA2*, *sul1*, *sul2*, *sul3*, *tet(A)*, and *dfrA12*] and mercury resistance genes were in a 189,237 bp IncC plasmid (pBL661) with a high sequence identity (87% coverage and 100% identity) to pUO-STmRV1 (CP018220), harbored in the *S*. 1,4,12:i:- Spanish clone (NZ_CP018220.1; strain LSP389-97). Pairwise comparison of pUO-STmRV1 and pBL661 (Figure 6) indicated that their genomes differed primarily in the absence of virulence genes *spvA*, *spvB*, *spvC*, *spvD*, and *spvR*, toxin-antitoxin *ccdA-B*, and the AMR gene *strA* in pBL661. BL661 was the only isolate of serotype *S*. 1,4,12:i:- harboring the *vagC* and *vagD* genes, both of which were present in pBL661. There were 140 SNP differences in the genomes of the two ST19 isolates BL661 and LSP389-97 containing the MDR plasmid, indicating evolution between these isolates, which possibly originated from the same progenitor.

### Phage lysis pattern for assessing host range

We determined the potential of phages to control the dissemination of our *Salmonella* isolates, including MDR and epidemic isolates. We studied the phage host range to ensure appropriate coverage against our panel of poultry isolates, using a host range “spot test” and phage killing assays at an MOI of 10 (Supplementary Table S8).

We noted differences in phage lysis patterns dependent on isolates, serovars, and phages (Table 4). The phage STW-77 showed the most efficacy against our collection of isolates, completely lysing 37.76% of the isolates tested (54 out of 143), and was the most effective for isolates originating from both countries. STW-77 activity, categorized according to *Salmonella* serotypes, indicated complete lysis of all of *S*. Stanley (6 of 6; 100%) and of *S*. 4,12:e,h:- (2 of 2; 100%) isolates. In addition, there was complete lysis of a high proportion of the remaining serovars: 83.33% of the *S*. 1,4,[5],12:i:- isolates; 80.95% of the *S*. Enteritidis isolates; 71.43% of the *S*. 1,4,12:i:- isolates; 66.67% of the *S*. Typhimurium isolates; 66.67% of the *S*. Agona isolates; and 50.00% of the *S*. Typhimurium var. Copenhagen isolates (Table 4).

**Table 4:** Rates of complete phage lysis activity according to serovars.

	Enteritidis	Typhimurium	1,4,12:i:-	1,4,[5],12:i:-	Agona	Schwarzengrund	Stanley	Typhimurium var. Copenhagen	6,8:e,h:-	4,12:e,h:-
SPFM9	80.95%	20.00%	42.86%	33.33%	–	–	33.33%	16.67%	–	50.00%
SPFM11	66.67%	20.00%	28.57%	33.33%	16.67%	–	33.33%	33.33%	–	50.00%
SPFM17	76.19%	26.67%	14.29%	–	16.67%	–	33.33%	50.00%	–	–
SPFM4	66.67%	33.33%	28.57%	16.67%	16.67%	–	33.33%	50.00%	–	50.00%
SPFM2	76.19%	40.00%	28.57%	16.67%	16.67%	–	33.33%	66.67%	–	50.00%
SPFM19	14.29%	–	–	–	–	–	–	–	–	–
SPFM14	61.90%	26.67%	28.57%	33.33%	16.67%	–	16.67%	–	–	50.00%
SPFM10	14.29%	–	–	–	–	–	–	–	–	–
SPFM12	14.29%	–	–	–	–	–	–	–	–	–
SPFM13	85.71%	40.00%	28.57%	16.67%	16.67%	–	33.33%	50.00%	–	–
SPFM20	71.43%	33.33%	28.57%	16.67%	16.67%	–	33.33%	33.33%	–	50.00%
SPFM1	76.19%	33.33%	28.57%	16.67%	16.67%	–	33.33%	33.33%	–	50.00%
SPFM3	76.19%	26.67%	28.57%	33.33%	16.67%	–	33.33%	33.33%	–	50.00%
SPFM16	80.95%	26.67%	28.57%	16.67%	16.67%	–	33.33%	50.00%	–	50.00%
SPFM15	71.43%	26.67%	28.57%	16.67%	16.67%	–	33.33%	33.33%	–	50.00%
SPFM7	66.67%	33.33%	28.57%	16.67%	16.67%	–	16.67%	50.00%	–	50.00%
SPFM6	76.19%	33.33%	28.57%	16.67%	16.67%	–	33.33%	50.00%	–	50.00%
SPFM8	80.95%	33.33%	28.57%	16.67%	16.67%	–	33.33%	50.00%	–	50.00%
SPFM21	71.43%	26.67%	28.57%	–	16.67%	–	33.33%	33.33%	–	50.00%
SPFM5	85.71%	33.33%	28.57%	–	16.67%	–	33.33%	33.33%	–	50.00%
STW-77	80.95%	66.67%	71.43%	83.33%	66.67%	33.33%	100.00%	50.00%	–	100.00%
SEW-109	42.86%	40.00%	28.57%	33.33%	–	–	66.67%	16.67%	25.00%	50.00%

The serovars against which the different phages were tested are shown. Serovars that were not completely lysed by the phages have been removed from the table, and a dash indicates no effect produced by the phage.

*S*. Enteritidis and *S*. Typhimurium are the two most clinically important serovars. Furthermore, *S*. Typhimurium and its monophasic variants *S*. 1,4,[5],12:i:- and *S*. 1,4,12:i:- were the serovars with the highest number of MDR isolates. The phages SPFM5 and SPFM13 were most effective against *S*. Enteritidis, completely lysing 85.71% of the isolates. On the other hand, phage STW-77 was the most effective against *S.* Typhimurium isolates and their monophasic variants *S*. 1,4,[5],12:i:- and *S*. 1,4,12:i:-, causing complete lysis in some, as described above. However, for *S*. Typhimurium var. Copenhagen, phage SPFM2 caused complete lysis in 66.67% of the isolates and was the highest in the phage panel. None of the phages in the panel induced complete lysis against the *S*. Mbandaka, *S.* Anatum, *S*. Corvallis, *S.* Virchow*, S*. Bovismorbificans, *S*. Hadar, and *S.* Kentucky isolates included in the study. In addition, STW-77 was the only phage in the panel that was more effective against MDR isolates (13 of 21; 61.90%) than against non-MDR isolates (15 of 32; 46.88%) and sensitive isolates (26 of 90; 28.89%; Supplementary Table S9).

We noted that no phage from the panel produced complete lysis against the MDR *S*. Kentucky ST198 clones BL700 and BL800. Phage STW-77, on the other hand, was successful in completely lysing 87.5% of the isolates identified as clones of the ST34 *S*. 1,4,[5],12:i:- European lineage. Furthermore, STW-77 and SEW-109 completely lysed isolate BL661, which harbored the MDR plasmid reported in a Spanish clone of the global lineage *S*. 1,4,[5],12:i:-.

## Discussion

Infections with *S*. enterica due to consumption of contaminated poultry products are among the most important causes of foodborne diseases worldwide. It is also well known that poultry farms are reservoirs of AMR *Salmonella* that affects humans (Chuanchuen et al., 2008; Liljebjelke et al., 2017). Therefore, we investigated 143 *Salmonella* isolates from Thai and UK poultry farms for their AMR burden and virulence genes using detailed genomic analysis.

Generally, numbers of AMR genes were low, with genes/mutations conferring resistance to sulfamethoxazole, ampicillin, and ciprofloxacin the most common. In fact, 27% of isolates collected from Thailand harbored *gyrA* mutations, making resistance to ciprofloxacin the highest in our panel of isolates. The levels of resistance in Thai and UK isolates generally concurred with previous reports of resistance in *Salmonella* from these countries, including the high levels of resistance for ciprofloxacin in Thailand, which has been rising worldwide in *Salmonella* (Weinberger and Keller, 2005; Chuanchuen et al., 2008; Directorate VM, 2014; Jain et al., 2020; Hengkrawit and Tangjade, 2022). From AMR characterization, serovars that reported the highest percentage of resistance to multiple antimicrobial classes were *S*. 1,4,[5],12:i:-, *S*. 1,4,12:i:-, *S*. Typhimurium, and *S*. Kentucky, as expected (Card et al., 2016; Zhang et al., 2018; Cadel-Six et al., 2021).

An extensive virulence determinant database constructed in this study in a manner similar to that for AMR genes (Duggett et al., 2016) enabled the linkage of virulence genes with serovar, country, and AMR profiles. We detected several serovars in which virulence genes were more likely to be present in MDR isolates, with some also associated with a particular country. For example, the virulence genes *safBCD*, *srfJ*, *lpfD*, and *fhuA* were present in both MDR *S*. Kentucky isolates, which were identified as clones of a ST198 *S*. Kentucky global lineage, although AMR genes were present on SGI-K and not linked to virulence. The presence of *lpfD* and *safBCD* has been shown previously (Hawkey et al., 2019), but as our virulence database was much larger, we additionally detected two important virulence genes: *fhuA*, which is involved in iron uptake (Wang et al., 2018); and *srfJ*, an effector in the *Salmonella* type III secretion system (Cordero-Alba et al., 2012). Another example included the detection of *sopD2*, which is involved in *S*. Typhimurium replication in the spleens of infected mice (Jiang et al., 2004) in all UK MDR *S*. Typhimurium, but variably present in Thai MDR *S*. Typhimurium. Therefore, several virulence genes were noticeably more common in MDR isolates, although they were not colocated with AMR genes. Nevertheless, these data suggest elasticity of these regions and the possible co-selection of virulence and AMR genes, which requires further study.

Resolving genomes using long reads and hybrid assemblies allowed further examination of selected isolates. It verified that the UK and Thailand *S*. Kentucky isolates included in our collections belonged to the MDR global lineage ST198, which has been reported worldwide, including in the UK and Thailand (Le Hello et al., 2011; Hawkey et al., 2019), suggesting that these clones are widespread in poultry.

The resolved genomes also showed that the eight *S*. Typhimurium and *S*. Typhimurium monophasic isolates from our UK and Thai panels belonged to another global epidemic clone, ST34 *S*. Typhimurium, and harbored SGI-4. The ST34 monophasic MDR variant of *S*. 1,4,[5],12:i:- is connected to human and animal infections (Hengkrawit and Tangjade, 2022) and has already been reported in the UK (Cadel-Six et al., 2021) and Thailand (Patchanee et al., 2020). Concerningly, not only is its prevalence increasing but an *mcr*-carrying variant conferring resistance to the last resort antibiotic colistin has been reported (Petrovska et al., 2016; Elnekave et al., 2018; Biswas et al., 2019; Patchanee et al., 2020; Ingle et al., 2021; Hengkrawit and Tangjade, 2022).

We were also able to characterize an IncC plasmid (pBL661) with high sequence identity to an MDR plasmid previously identified in a Spanish *S*. 1,4,12:i:- clone (García et al., 2011). The MDR and virulence characteristics of this plasmid are thought to have contributed to its epidemiological success (García et al., 2011), and the toxin-antitoxin *vagCD* system is likely connected to its maintenance (Han et al., 2012) and global spread (Duprilot et al., 2017).

Therefore, the presence of multiple MDR *Salmonella* epidemic clones in our collection supports the perception that they are spreading worldwide, posing a public health risk and challenging current treatment options to control foodborne infections. Given the prolonged and arduous process of developing new antibiotics (O’Neil, 2014; Romero-Calle et al., 2019; Nale et al., 2021), we explored bacteriophages as a viable alternative for controlling *Salmonella* dissemination. The phages were tested on *Salmonella* from UK and Thailand, and overall showed good coverage against the panel tested, highlighting their activity against clinically important serovars such as *S*. Enteritidis and *S*. Typhimurium and its variants. Also, phage STW-77 successfully completely lysed most isolates identified as clones of the ST34 *S*. 1,4,[5],12:i:- European lineage. Furthermore, STW-77 and SEW-109 completely lysed the isolate that harbored pBL661, the Spanish MDR plasmid of the global lineage *S*. 1,4,12:i:-.

These phages have already been used successfully in phage cocktails against *Salmonella* in *in vivo* analyses in *Galleria mellonella* (Nale et al., 2021) and mice (Sukjoi et al., 2022), as well as in *ex vivo* analyses in avian, porcine, and human epithelial cell cultures (Nale et al., 2023); therefore, their use on poultry farms as biocontrols is likely to be the next step. Unfortunately, no phage tested in this study completely lysed the *S*. Kentucky ST198 MDR clones; therefore, further study is needed.

## Conclusion

*Salmonella* linked to poultry sources can cause gastrointestinal infections in humans due to the presence of virulence genes that can cause infections. The presence of different MDR clones of *Salmonella* with a plethora of virulence genes in isolates collected from poultry in the UK and Thailand supports the notion of a global increase. The work performed in this study provides examples of types of characterizations that can be undertaken for the genomic surveillance of *Salmonella* to accurately identify its spread, especially of global epidemic clones that may be present on different farms worldwide, and our results indicate the potential of phage-based biocontrol to mitigate their transmission.

## Author’s summary

Consuming *Salmonella-*infected poultry products is a significant source of gastrointestinal infection, causing more than 100,000 human deaths annually worldwide. The pathogens may also harbor antimicrobial resistance (AMR) genes and be multidrug resistant, making treatment difficult. We characterized 143 isolates from poultry from UK and Thai farms to determine diversity in the AMR and virulence genes they harbored. Additionally, we established the sensitivity of the isolates to a panel of bacteriophages. The results indicated genes harboring resistance to antimicrobials differed between the countries, possibly due to differing farming practices; however, 14‒15% of all isolates were multidrug resistant. The presence of virulence genes showed some variation, with several correlating to the resistance status of isolates. Further exploration using long-read sequencing revealed that multiple isolates from both countries were global epidemic MDR clones, supporting the notion of a global increase. From the panel of bacteriophages tested, phage STW-77 was the most effective in lysing isolates and demonstrated that phage-based biocontrol has the potential to control dissemination in poultry flocks of *Salmonella* associated with human infections, including global epidemic clones, which may subsequently enter the food chain.

## Data availability statement

The datasets presented in this study can be found in online repositories. The names of the repository/repositories and accession number(s) can be found at: https://www.ncbi.nlm.nih.gov/, SUB12515972, PRJNA919003.

## Author contributions

MA, MC, SK, and DM: initial concept. AL-G, MAO, EG, and JN: laboratory studies. AL-G, MAO, and JN-G: data analysis. AL-G, MAO, JN-G, JN, EG, PP, CS, PT, DM, SK, MC, and MA: Manuscript writing, review, and finalizing. All authors contributed to the article and approved the submitted version.

## Funding

This work was funded by Biotechnology and Biological Sciences Research Council (BBSRC), grant number RM38G0140 awarded to MC and the National Science and Technology Development Agency (NSTDA), grant number P-18-50454 awarded to SK.

## Conflict of interest

The authors declare that the research was conducted in the absence of any commercial or financial relationships that could be construed as a potential conflict of interest.

## Publisher’s note

All claims expressed in this article are solely those of the authors and do not necessarily represent those of their affiliated organizations, or those of the publisher, the editors and the reviewers. Any product that may be evaluated in this article, or claim that may be made by its manufacturer, is not guaranteed or endorsed by the publisher.

## Footnotes

^1^ https://britishpoultry.org.uk/british-poultrys-successful-stewardship-on-antibiotic-use/
