# Corrigendum: Pathogen genomics and phage-based solutions for accurately identifying and controlling *Salmonella* pathogens

**DOI:** 10.3389/fmicb.2023.1221779

**Published:** 2023-08-08

**Authors:** Angela V. Lopez-Garcia, Manal AbuOun, Javier Nunez-Garcia, Janet Y. Nale, Edouard E. Gaylov, Preeda Phothaworn, Chutikarn Sukjoi, Parameth Thiennimitr, Danish J. Malik, Sunee Korbsrisate, Martha R. J. Clokie, Muna F. Anjum

**Affiliations:** ^1^Department of Bacteriology, Animal and Plant Health Agency, Weybridge, United Kingdom; ^2^Department of Veterinary and Animal Science, Scotland's Rural College, Inverness, United Kingdom; ^3^Department of Genetics and Genome Biology, University of Leicester, Leicester, United Kingdom; ^4^Department of Immunology, Faculty of Medicine Siriraj Hospital, Mahidol University, Bangkok, Thailand; ^5^Department of Microbiology, Faculty of Medicine, Chiang Mai University, Chiang Mai, Thailand; ^6^Department of Chemical Engineering, Loughborough University, Loughborough, United Kingdom

**Keywords:** antimicrobial resistance, *Salmonella*, virulence genes, genomics, bacteriophages, serovar

In the published article, there was an error in the Figure 3 and its legend as published. Figure 3B was removed from the manuscript; however, the associated legend text still needs to be removed and the key was not completely included in the original picture. The corrected Figure 3 and its caption appear below.

**Figure 3:**
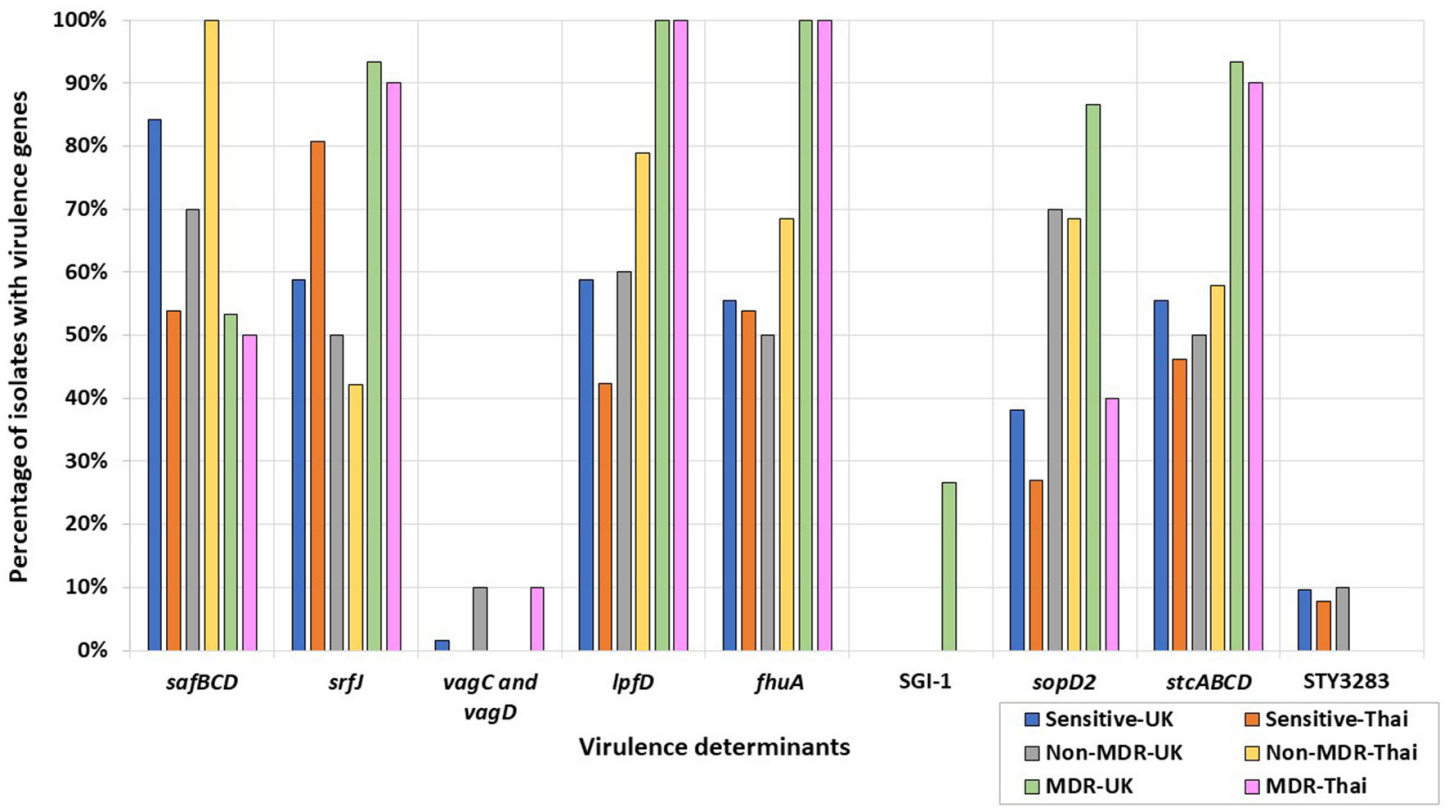
Percentages of UK and Thai MDR, non-MDR, and sensitive isolates harboring virulence determinants. MDR isolates from the UK (green), non-MDR isolates from the UK (grey), sensitive isolates from the UK (blue), MDR isolates from Thailand (pink), non-MDR isolates from Thailand (yellow), and sensitive isolates from Thailand (orange) have been included.

In the published article, there was an error in Table 1 as published. *strA, strB* and *tetA(B)* genes were misspelt as *straA, straB* and *tet-AB*. The corrected Table 1 and its caption appear below.

**Table 1:** AMR genotypes of 143 isolates identified by the APHA Seqfinder.

**Classes**	**Antimicrobial**	**Genes**	**UK (*n* isolates)**	**Percentage**	**Thai (*n* isolates)**	**Percentage**
Penicillin	Ampicillin	*bla_*CARB*−2_*	4 of 88	4.55%	0 of 55	0%
		*bla_*TEM*−135_*	0 of 88	0%	3 of 55	5.45%
		*bla_*TEM*−1*b*_*	11 of 88	12.50%	13 of 55	23.64%
		*bla_*TEM*−1*D*_*	1 of 88	1.14%	0 of 55	0%
Macrolides	Azithromycin	*mphB*	1 of 88	1.14%	0 of 55	0%
Phenicol	Chloramphenicol	*cmlA1*	4 of 88	4.55%	1 of 55	1.82%
		*floR*	4 of 88	4.55%	0 of 55	0%
Fluoroquinolone	Ciprofloxacin	*mutation gyrA*	4 of 88	4.55%	15 of 55	27.27%
		*qnrS1*	0 of 88	0%	4 of 55	7.27%
	Nalidixic acid	*mutation gyrA*	4 of 88	4.55%	15 of 55	27.27%
Aminoglycoside	Gentamicin	*aac(3)-Id*	1 of 88	1.14%	0 of 55	0%
		*aac(3)-IVa*	2 of 88	2.27%	1 of 55	1.82%
	Streptomycin	*strA*	7 of 88	7.95%	7 of 55	12.73%
		*strB*	9 of 88	10.23%	7 of 55	12.73%
Sulfonamide	Sulfamethoxazole	*sul1*	4 of 88	4.55%	1 of 55	1.18%
		*sul2*	6 of 88	6.82%	8 of 55	14.55%
		*sul3*	7 of 88	7.95%	5 of 55	9.09%
Tetracycline	Tetracycline	*tetA(B)*	7 of 88	7.95%	5 of 55	9.09%
		*tet(A)*	6 of 88	6.82%	2 of 55	3.64%
		*tet(G)*	4 of 88	4.55%	0 of 55	0%
		*tet(M)*	0 of 88	0%	1 of 55	1.82%
Diaminopyrimidine	Trimethoprim	*dfrA12*	4 of 88	4.55%	1 of 55	1.82%

AMR genes were grouped according to antimicrobial classes, and they are shown alongside the percentage of isolates per country harboring the AMR gene.

In the published article, the reference for the virulence genes *safBCD, srfJ, lpfD*, and *fhuA* were present in both MDR *S*. Kentucky isolates, which were identified as clones of a ST198 *S*. Kentucky global lineage (Martínez and Baquero, 2002; Beceiro et al., 2013) was incorrectly written as (Martínez and Baquero, 2002; Beceiro et al., 2013). It should be deleted.

In the published article, there was an error to **Materials and methods**, *Phylogenetic and SNP analysis*. The reference name should not contain commas.

This sentence previously stated:

“Snippy version v4.6.0 (Seemann, 2015) was used to detect SNPs in the core genome of *S*. Kentucky isolates BL700 and BL800, and two *S*. Kentucky MDR ST198 isolates from earlier research [SAMN08784244 and SAMN08784253; (Hawkey et al., 2019)], aligning them against the reference 201,001,922 (CP028357).”

The corrected sentence appears below:

“Snippy version v4.6.0 (Seemann, 2015) was used to detect SNPs in the core genome of *S*. Kentucky isolates BL700 and BL800, and two *S*. Kentucky MDR ST198 isolates from earlier research [SAMN08784244 and SAMN08784253; (Hawkey et al., 2019)], aligning them against the reference 201001922 (CP028357).”

In the published article, there was an error in **Results**, *Antimicrobial resistance characterization*. The Thai *S*.1,4,12:i:- isolate does not present resistance to streptomycin.

This sentence previously stated:

“One Thai isolate, serotyped as *S*. 1,4,12:i:-, was resistant to seven antimicrobial classes (ampicillin, chloramphenicol, ciprofloxacin-nalidixic acid, gentamicin-streptomycin, sulfamethoxazole, tetracycline, and trimethoprim).”

The corrected sentence appears below:

“One Thai isolate, serotyped as *S*. 1,4,12:i:-, was resistant to seven antimicrobial classes (ampicillin, chloramphenicol, ciprofloxacin-nalidixic acid, gentamicin, sulfamethoxazole, tetracycline, and trimethoprim).”

In the published article, there was an error in **Results**, *Characterization of MDR isolates with distinct virulence profiles*, 3rd Paragraph. The *tetA(B)* gene was misspelt.

This sentence previously stated:

“From the resolved genome of MDR isolate BL708 *S*. 1,4,[5],12:i:-, we identified SGI-4 in the chromosome with genes showing resistance to copper, arsenic, mercury, and antimicrobials [*bla*_*Tem*−1*b*_, *sul2, tet(AB), strA, strB*].”

The corrected sentence appears below:

“From the resolved genome of MDR isolate BL708 *S*. 1,4,[5],12:i:-, we identified SGI-4 in the chromosome with genes showing resistance to copper, arsenic, mercury, and antimicrobials [*bla*_*TEM*−1*B*_, *sul2, tetA(B), strA, strB*].”

In the published article, there was an error with the percentage of MDR isolates reported in the **Author's Summary**.

The sentence previously stated:

“The results indicated genes harboring resistance to antimicrobials differed between the countries, possibly due to differing farming practices; however, 17–18% of all isolates were multidrug resistant.”

The corrected sentence appears below:

“The results indicated genes harboring resistance to antimicrobials differed between the countries, possibly due to differing farming practices; however, 14%−15% of all isolates were multidrug resistant.”

In the published article, there was an error in **Supplementary Table S5**. The *tet-AB* gene was misspelt and should be replaced by *tetA(B)*. The supplementary table with the error typo amended has been submitted.

The authors apologize for these errors and state that this does not change the scientific conclusions of the article in any way. The original article has been updated.
